# The Effect of an Accelerator on Cement Paste Capillary Pores: NMR Relaxometry Investigations

**DOI:** 10.3390/molecules26175328

**Published:** 2021-09-02

**Authors:** Ioan Ardelean

**Affiliations:** Physics and Chemistry Department, Technical University of Cluj-Napoca, 400114 Cluj-Napoca, Romania; ioan.ardelean@phys.utcluj.ro

**Keywords:** NMR relaxometry, cement hydration, accelerators, pore evolution, partially saturated, fractal dimension

## Abstract

Nuclear Magnetic Resonance (NMR) relaxometry is a valuable tool for investigating cement-based materials. It allows monitoring of pore evolution and water consumption even during the hydration process. The approach relies on the proportionality between the relaxation time and the pore size. Note, however, that this approach inherently assumes that the pores are saturated with water during the hydration process. In the present work, this assumption is eliminated, and the pore evolution is discussed on a more general basis. The new approach is implemented here to extract information on surface evolution of capillary pores in a simple cement paste and a cement paste containing calcium nitrate as accelerator. The experiments revealed an increase of the pore surface even during the dormant stage for both samples with a faster evolution in the presence of the accelerator. Moreover, water consumption arises from the beginning of the hydration process for the sample containing the accelerator while no water is consumed during dormant stage in the case of simple cement paste. It was also observed that the pore volume fractal dimension is higher in the case of cement paste containing the accelerator.

## 1. Introduction

Reducing the carbon footprint associated with cement production is an important objective nowadays, and it can be achieved by a better exploitation of the cement composites, for instance, using 3D printing technology [1]. Building without formworks has the advantage of saving cost, time and materials associated with formwork construction. However, it also implies some significant materials engineering challenges to substitute all the requirements which are typically fulfilled by the formwork. One of the most important characteristics of concrete extrusion, opposite to castable concrete, is that it requires fast-setting and low slump [2]. These requirements must be fulfilled because the material is unsupported after leaving the print nozzle. That is why the cement-based materials for 3D printing applications are designed to exhibit fast build-up process. Moreover, it is necessary that the mix energy used to break the bounds inside the material should be low to enable delivery by normal pumps [1].

The speed of strength development of cement mixtures is controlled by adding accelerators [3,4,5] or retarders [5,6,7] but also admixtures such as silica fume [8] or carbon nanotubes [9]. Note, however, that choosing the correct type and amount of accelerator, retarder or admixture is a difficult task. On the one hand, the rapid strength development allows building of more layers on top of one another, and on the other hand, it reduces the building time. Moreover, long setting times would be necessary to keep the surface of the layers chemically active to form interfaces between layers of which behavior is close to the bulk material [2,5,10,11]. Consequently, hydration kinetics must be controlled in an accurate manner so that the material does not set during the printing process but, instead, right after deposition to support its own weight and that of subsequently deposited layers of material. There is a so-called “open time” [5,10] during which a specified volume of material must be extruded in the 3D printing process. In practice, the open time overlaps with the dormant stage of the hydration process [5]. The open time is influenced by the cement sample constituents and the temperature [12]. Controlling the open time for cement mixtures is essential for successful 3D printing applications. That is why the development of new approaches to monitor the hydration process is an important issue.

Low-field nuclear magnetic resonance (NMR) relaxometry techniques are valuable instruments for monitoring the cement hydration under the influence of different additives and admixtures [6,7,8,13,14,15,16,17,18,19]. They are completely noninvasive, do not require any special sample preparation and can be applied even during the hydration process. By using NMR relaxometry techniques it is possible to monitor the pore evolution and water consumption during the hydration process. Note, however, that when applying NMR relaxometry to cement-based materials, during the hydration, it is arbitrarily assumed that the pores are fully saturated with liquid, an assumption which was never demonstrated but continues to be used in the literature. Here, this assumption will be disregarded, and the data will be analyzed in a more general manner. The results of the new approach will be compared with those based on the pore volume fractal dimension analysis [20,21,22,23,24].

## 2. Theoretical Background

### 2.1. The Process of Cement Hydration

The cement hydration process starts immediately after mixing the cement grains with water molecules [25]. It produces not only a simple neutral colloidal gel where the cement grains are dispersed in water, but also some internal organization arises [25]. This is because some amount of water almost instantly combines with the cement grains producing micro-organized systems such as flocculation of cement grains, chemical reactions, ettringitic pores and so on [25]. Water inside these pores was called “embedded water” and is characterized by a shorter transverse relaxation time in NMR relaxometry [19]. The remaining water, filling in the empty space between these microstructures, represents the “capillary water” and has a longer relaxation time [19]. Note that in the present work, the cement chemistry abbreviations are used, where C = CaO, S = SiO_2_, A = Al_2_O_3_, F = Fe_2_O_3_ and H = H_2_O [25].

It is customary to separate the hydration process of cement paste into five stages: the initial stage, the dormant stage, the hardening stage, the cooling stage and the densification stage [12,19,26]. These hydration stages were extensively discussed in the literature both with respect of their duration and the influences introduced by different experimental parameters [6,19,25]. Here, we will only shortly describe them to understand the pore development. Thus, during the initial stage (less than 15 min) the C3A component of the clinker reacts with water and releases heat. The ettringite formation starts immediately creating a layer around the cement grains that isolates the paramagnetic relaxation centers (Fe^3+^) on the surface of cement grains from the bulk water thus reducing the relaxation rate [6,15,27]. During the dormant stage (between 15 min and 2 h), the silicates (C3S and C2S) dissolve in water and the calcium and hydroxide ions are slowly released into the solution. No changes in the porosity and no increase of the ettringite layer are expected during this stage. During the hardening stage (between 2 h and 12 h), the hydroxide and calcium ions reach a critical concentration and the calcium silicate hydrate (C-S-H) and calcium hydroxide (CH) begin the crystallization process. Furthermore, during this period the development of the ettringite layer continues. In the cooling period (between 12 h and 20 h), the reaction of C3S is much slower because the C-S-H and CH restricts the contact between water and unhydrated cement grains. However, the porosity reduces, and the relaxation time decreases accordingly. The densification stage lasts from 20 h to the end of the cement hydration (conventionally considered 28 days). During this period C-S-H and CH form a solid mass; this produces an increase in the strength and durability of cement paste and, at the same time, a decrease in the permeability. The slow formation of hydrate products occurs and continues providing that water and unhydrated silicates are present.

### 2.2. NMR Relaxation in Partially Saturated Pores

In the NMR relaxometry of porous materials it is routinely considered that the pores are saturated with the filling liquid and the observed transverse relaxation rate of confined molecules is a weighted average between the bulk relaxation rate and the surface relaxation rate of molecules confined inside a thin layer of few molecular diameters, uniformly covering the internal surface of the pores [16,28]. However, there are many situations when the pores are only partially saturated with the liquid. In that case, the relaxation rate depends on the pore filling and the liquid distribution on the pore surface [16,28,29,30]. Assuming that the confined molecules wet the surface of the pores, the relaxation rate can be expressed as a weighted average between the relaxation rate of the remaining bulk-like liquid and the surface relaxation rate. In the case of cement-based materials, the bulk-like contribution can be neglected, and the relaxation rate can be approximated as

(1) $$\frac{1}{T_{2}}=\rho \frac{S_{p}}{V_{l}}.$$

where $S_{p}$ is the pore surface and $V_{l}$ is the liquid volume inside the pores, under partially saturated conditions. The constant ρ is called relaxivity and depends on pore surface properties, filling molecules and the intensity of the magnetic field of the experiment [15,18,27,30]. In the case of saturated pores, $V_{l}=V_{p}$, where $V_{p}$ is the pore volume. Provided that relaxivity of the surface is known, for saturated pores it is possible to determine the pore size distribution from relaxation time distribution measurements. Note that in all the investigations reported in the literature related to relaxation studies on cement hydration, it is a priori assumed that $V_{l}=V_{p}$. In the present work, this assumption is eliminated, and the data are evaluated based on Equation (1) where only the volume of the confined liquid is considered. Consequently, by representing the ratio $V_{l}/T_{2}$ as a function of hydration time, information on surface evolution during the hydration can be extracted. This approach will be exploited here to monitor the surface evolution of the capillary pores in cement paste during the first hours of hydration (dormant and hardening stage).

### 2.3. The Transverse Relaxation Time and the Fractal Dimension

Starting with the introduction of fractals by Mandelbrot in 1977 [31], the geometrical structure of pores and the pore surface could be described based on fractal dimension [20,21,22]. The concept of fractal dimension can be used also for analyzing a volume distribution of pores [22]. Thus, a uniform pore size distribution corresponds to the topological dimension of three while a variation in the pore size distribution can be described by a fractal volume dimension $D_{f}<3$. From the practical side, it was demonstrated that, in the case of fiber recycled concrete, there is a linear relationship between the pore volume fractal dimension and the strength [21].

The basic theory that relates the fractal geometry of the porous structure to the NMR relaxation data is comprehensively described by Zhang and Weller [22]. They have shown that the transverse relaxation time of molecules confined inside porous structures can be related to the pore volume fractal dimension, $D_{f}$, in accordance with the work in [22]:

(2) $$\log\left(V_{c}\right)=\left(3-D_{f}\right)\log\left(T_{2}\right)-\left(3-D_{f}\right)\log\left(T_{2}^{\max D}\right)$$

where $V_{c}$ is the cumulative volume fraction of the wetting fluid in the pore space, with the relaxation time smaller than $T_{2}$. It is defined as the ratio between the volume of pores characterized by a relaxation time smaller than $T_{2}$ and the total pore volume. The cumulative volume can be calculated from NMR relaxation time distribution by dividing the area under the curve, obtained for relaxation times smaller than $T_{2}$, to the total area of the distribution [22]. $T_{2}^{\max D}$ represents the maximum detectable relaxation time in the relaxation time distribution. Note that the above formula was derived under the condition $T_{2}>>T_{2}^{\min D}$, where $T_{2}^{\min D}$ is the minimum relaxation time detected in the distribution. That is why fitting of cumulative volumes with a linear curve to extract the slope and thus to determine the pore volume fractal dimension is only possible under such circumstances [22]. Consequently, the data will be fitted here only for relaxation values close to the $T_{2}^{\max D}$. The above Equation (2) originates in the assumption that the pore dimension is proportional with the relaxation time. In the case of partially saturated pores, the above equation is still valid, but the probed dimension refers there to the liquid volume inside the pore space.

## 3. Results and Discussion

**Figure 1:**
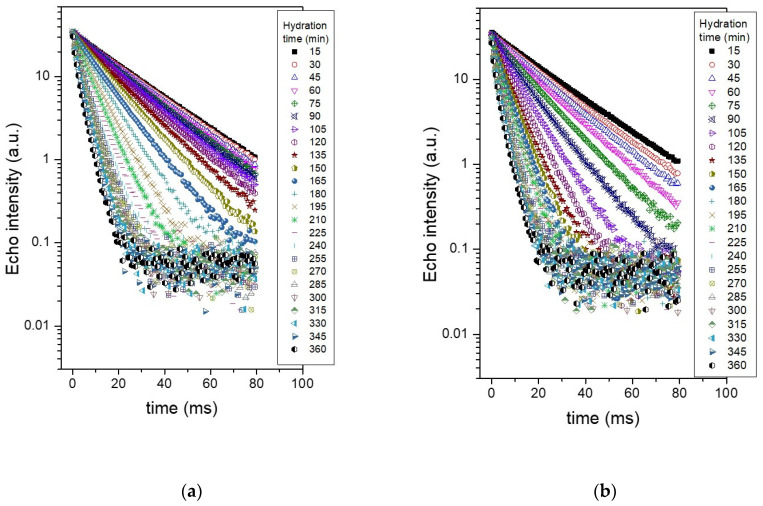
CPMG echo trains recorded for the two samples, at different hydration times, as indicated in the legend: (**a**) Cement paste sample prepared at a water-to-cement ratio of 0.4; (**b**) Cement paste prepared at 0.4 water-to-cement ratio which additionally contains 3% accelerator (Ca(NO_3_)_2_).

The samples under investigation were the simple cement paste (CP) prepared with a water-to-cement ratio of 0.4 and a cement paste additionally containing 3% by cement weight of Ca(NO_3_)_2_, as accelerator. The echo trains recorded in the Carr–Purcell–Meiboom–Gill (CPMG) [32,33] experiments, performed at 15 min intervals, during the first 6 h of hydration are shown in Figure 1. Comparing the time evolution of the two samples it is observed a faster increase in the slope of the echo trains recorded for the sample containing the accelerator (Figure 1b). This effect arises as calcium nitrate increases the concentration of calcium ions leading to a faster supersaturation with respect to the silicate hydrates [5]. This in turn produces a faster development of the C-S-H phase and faster consumption of the capillary water in the hydration process (see Section 2.1 above).

**Figure 2:**
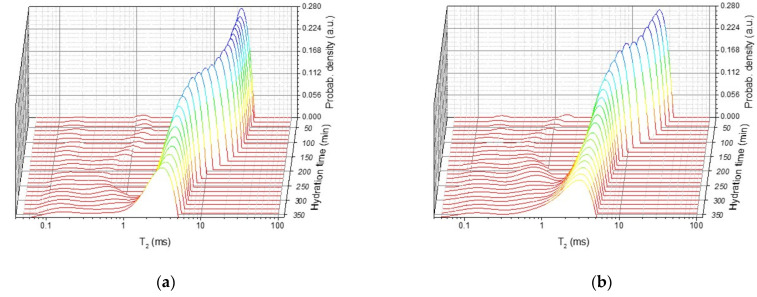
Relaxation time distributions for the two samples, at different hydration times, as indicated in the legend: (**a**) Cement paste sample prepared at a water-to-cement ratio of 0.4; (**b**) Cement paste prepared at 0.4 water-to-cement ratio which additionally contains 3% accelerator (Ca(NO_3_)_2_), by cement mass.

To monitor the effects of water consumption and the evolution of capillary pores the curves depicted in Figure 1 can be analyzed using a numerical Laplace inversion [34,35]. The numerical analysis provides the relaxation time distributions shown in Figure 2. One can observe three peaks corresponding to different water reservoirs inside the sample: two peaks of smaller area and one peak of larger area. The first and the second peak (from the left) can be attributed to the water inside intra- and inter C-S-H pores, respectively [14,15,16,27]. They arise immediately after mixing the cement grains with water molecules and remain constant during the dormancy stage. The shift in the position of the second peak to smaller values can be attributed to a denser inter C-S-H phase [19]. Beginning with the acceleration stage, the area of the two peaks starts to increase showing that more and more intra and inter C-S-H pores are formed inside the sample.

**Figure 3:**
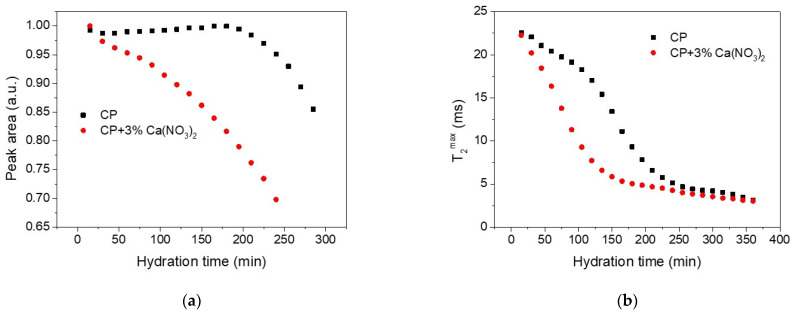
(**a**) The peak area evolution during hydration for the capillary pores of the two samples; (**b**) Evolution of the relaxation time corresponding to the peak maximum during the hydration.

The third peak (the largest one) in the relaxation time distributions, shown in Figure 2, corresponds to the capillary water contained between the cement grains during the first stage of hydration and will be monitored here in more detail. The area of the peak is proportional with the amount of water inside the capillary pores. Figure 3 shows the dependence of the peak area (Figure 3a) and of the position of the peak maximum (Figure 3b) on the hydration time in the case of the simple cement paste (CP) and the cement paste containing the accelerator (CP + 3% Ca(NO_3_)_2_). One can observe faster decay of the peak area and of $T_{2}^{\max}$ in the case of sample containing the accelerator as compared with the simple cement paste. The faster evolution demonstrates accelerated hydration dynamics introduced by the calcium nitrate. This observation is consistent with the previous reports that calcium nitrate increases the concentration of calcium ions, leading to a faster supersaturation with respect to the silicate hydrates and thus producing a faster development of the C-S-H phase and faster consumption of the capillary water in the hydration process [5].

**Figure 4:**
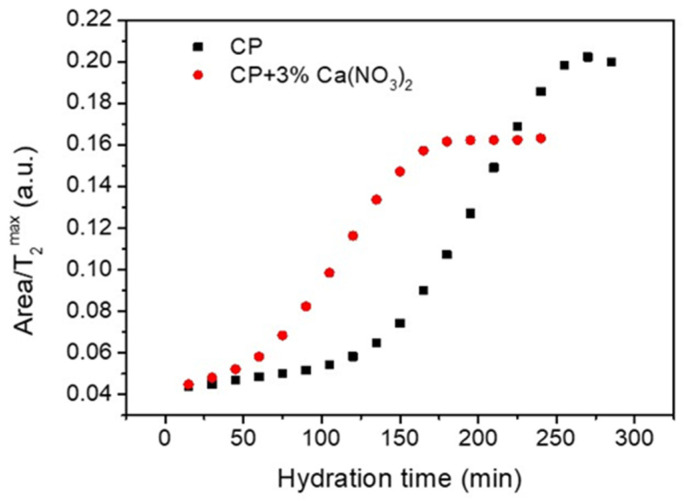
Ratio between the peak area and the peak maximum in the relaxation time distributions shown in Figure 2 for the capillary water component of the two samples, as indicated in the legend. In the case of sample containing the accelerator, the evaluation was restricted to shorter relaxation times due to the overlapping of the capillary peak with the signal corresponding to the C-S-H pores.

Comparing the area evolution of the capillary water peak for the two samples (Figure 3a) it is observed that the area of CP peak remains constant up to 3 h of hydration, but a continuous decrease arises in the case of cement paste containing the accelerator (CP + 3% Ca(NO_3_)_2_). The relatively constant peak area of the CP capillary pores indicates that the pore volume does not change during the dormancy stage and water is not consumed to form hydration products. Consequently, the reduction of the relaxation time observed in Figure 3b during the dormant stage can only be attributed to the changes in the pore surface. To demonstrate this conclusion, we notice from Equation (1) that if we represent the ratio $V_{l}/T_{2}^{\max}$ as a function of hydration time, this ratio will describe the pore surface evolution. Note that $V_{l}$ is proportional with the peak area, consequently $Area/T_{2}{}^{\max}$ is represented in Figure 4 as a function of hydration time. According to Equation (1), this representation is independent of the assumption that the pores are saturated with water and provides information of pore surface evolution during the hydration. As one can observe, the surface of capillary pores increases for both samples, but the process is faster for the sample containing the accelerator (circles).

**Figure 5:**
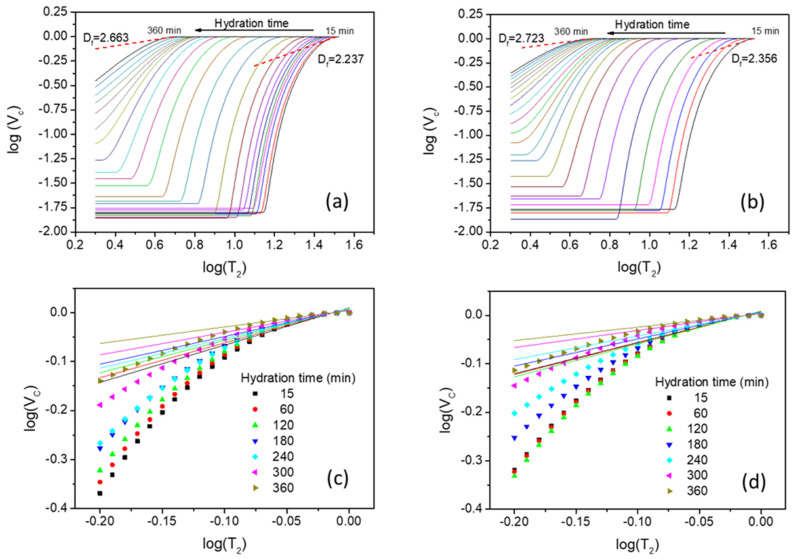
Versus $\log\left(T_{2}\right)$ at different hydration times for the capillary water peak in Figure 2 in the case of cement paste sample (**a**) and the cement paste containing the accelerator (**b**). $T_{2}$ was considered here in milliseconds. The linear fits with Equation (2) for the two samples were performed after shifting the data on the abscissa axis, for a direct comparison. The same region, between −0.08 and 0.00 on the abscissa axis, was used for all fits. A continuous change in the slope is observed both for the simple cement paste (**c**) and the sample containing the accelerator (**d**). The coefficients of determination $R^{2}$ in all fits were bigger than 0.93, indicating a good linear approximation.

The changes in the pore morphology can be also described by monitoring the volume fractal dimension $D_{f}$. This quantity can be evaluated based on the log-log representation suggested by Equation (2). The $\log\left(V_{c}\right)$ versus $\log\left(T_{2}\right)$ curves are shown in Figure 5 for the two samples during the first six hours of hydration. A decrease in the slope for both samples is obtained during the hydration which is equivalent, based on Equation (2), with an increase in the fractal dimension. As can be observed from the figure, the fractal dimension varies from 2.237 to 2.663 in the case of simple cement paste (Figure 5a), and from 2.356 to 2.723 in the case of cement paste containing the accelerator (Figure 5b). This variation in fractal dimension is continuous (see the slopes in Figure 5c,d, respectively) and correlated with the change in the surface size revealed in Figure 4. Note that, the increase in the fractal dimension of cement based materials was associated with an increase in their strength [21]. Here, the increase in fractal dimension could be again correlated with the increase in strength during the hydration. However, establishing a direct relationship between compressive strength and pore volume fractal dimension, determined by NMR, requires supplemental investigations.

## 4. Materials and Methods

### 4.1. Sample Preparation

Two samples, with the same water-to-cement ratio of 0.4, were prepared and comparatively investigated in the present study. One sample is a pure cement paste (CP) obtained by mixing Portland cement with water and the other additionally contains 3% Ca(NO_3_)_2_ by cement mass. The two samples were prepared with white Portland cement CEM I 52.5 R (Holcim, Bucuresti, Romania), fulfilling the European Standard BS EN 197-1. The white cement was chosen here on purpose due to its low content of iron oxide (<0.5%), in order to reduce internal gradients that can be induced by susceptibility difference between the solid matrix and filling liquid [13,36]. The Ca(NO_3_)_2_ accelerator was acquired from NORDIC Chemicals SRL, Cluj-Napoca, Romania. Before mixing with cement powder, the accelerator was dissolved in water. The ingredients were then mixed for 5 min using a mixer, at 100 rpm. The resulting paste was poured into 10 mm glass tubes and then introduced inside the probe head. The tubes were sealed to prevent water evaporation. The first NMR measurements were always performed at 15 min counting from the initiation of the mixing process and the last after 6 h of hydration.

### 4.2. NMR Measurements

Transverse relaxation measurements of fluids confined inside the cement paste pores were performed using the well-known Carr–Purcell–Meiboom–Gill (CPMG) technique [32,33]. In the CPMG pulse sequence, an initial 90° radiofrequency pulse around the *y*-axis is followed by a train of 180° pulses around the *x*-axis at time instants τ, 3τ, 5τ, … The attenuation of the echo train recorded at the time instants 2τ, 4τ, 6τ, … contains information about the transverse relaxation time distribution inside the sample. If the sample is heterogeneous and the echo train attenuation is multiexponential, a numerical Laplace inversion [34,35] of the echo train provides the relaxation time distribution. In the case of liquids confined inside porous media, the relaxation time distribution mimics the pore size distribution and a quantitative description of the pore sizes can be obtained provided that the relaxivity of the pore surface is known from independent measurements. The main advantage of such a multiple echo technique for the determination of the transverse relaxation time is that it allows fast multiple accumulations of the echo train signal—an important issue in increasing the detection sensitivity. Furthermore, due to the short echo times implemented it reduces diffusion effects on transverse relaxation measurements [13,36].

The experiments were performed using a Bruker Minispec MQ20 instrument (Bruker BioSpin GmbH, Rheinstetten, Germany), operating at a proton resonance frequency of 20 MHz. The CPMG echo trains consisting of 1000 echoes were recorded after 32 scans, with an echo time of 80 µs and a recycle delay of 0.5 s. With these parameters, the recording duration of one echo train was short enough to prevent sample changes during the experiment. The measurements were performed at 35 °C, the working temperature of the Bruker Minispec MQ20 instrument, without using the external temperature control unit.

## 5. Conclusions

Monitoring the evolution of NMR relaxation time under the influence of different parameters can be used as a tool in determining the pore evolution of cement-based materials. Here, the influence of an accelerator on the surface evolution of capillary pores was studied using a low-field NMR instrument. The approach employed here removes the generally used assumption that the capillary pores are saturated with water during the early hydration. Based on the new approach, it was shown that the surface of capillary pores increases during the early hydration (less than 6 h) even during the dormant stage and this effect is higher in the presence of an accelerator. However, in the case of simple cement paste, the pore volume sems to be constant during the dormant stage, and a clear identification of the dormant stage is possible by plotting the area of the peak versus hydration time. In the presence of an accelerator there is a continuous consumption of water in capillary pores, and one cannot clearly identify the dormant stage, at least for the accelerator content and hydration temperature used in the experiments. The volume fractal dimension of capillary pores increases during the hydration in the case of both samples, with higher values in the case of cement paste containing the accelerator. This indicates a more uniform pore distribution in the case of the sample containing the accelerator.

## Data Availability

Not applicable.

## Sample Availability

Samples of the compounds are not available.
